# The Added Diagnostic Value of Dynamic Contrast-Enhanced MRI at 3.0 T in Nonpalpable Breast Lesions

**DOI:** 10.1371/journal.pone.0094233

**Published:** 2014-04-08

**Authors:** Laura G. Merckel, Helena M. Verkooijen, Nicky H. G. M. Peters, Ritse M. Mann, Wouter B. Veldhuis, Remmert K. Storm, Teun Weits, Katya M. Duvivier, Thijs van Dalen, Willem P. Th. M. Mali, Petra H. M. Peeters, Maurice A. A. J. van den Bosch

**Affiliations:** 1 Department of Radiology, University Medical Center Utrecht, Utrecht, The Netherlands; 2 Imaging division, University Medical Center Utrecht, Utrecht, The Netherlands; 3 Department of Radiology, Radboud University Medical Center, Nijmegen, The Netherlands; 4 Department of Radiology, Albert Schweitzer Hospital, Dordrecht, The Netherlands; 5 Department of Radiology, Diakonessen Hospital, Utrecht, The Netherlands; 6 Department of Surgery, Diakonessen Hospital, Utrecht, The Netherlands; 7 Julius Center for Health Sciences and Primary Care, University Medical Center Utrecht, Utrecht, The Netherlands; 8 Department of Radiology, VU University Medical Center, Amsterdam, The Netherlands; The Norwegian University of Science and Technology (NTNU), Norway

## Abstract

**Objective:**

To investigate the added diagnostic value of 3.0 Tesla breast MRI over conventional breast imaging in the diagnosis of *in situ* and invasive breast cancer and to explore the role of routine versus expert reading.

**Materials and Methods:**

We evaluated MRI scans of patients with nonpalpable BI-RADS 3–5 lesions who underwent dynamic contrast-enhanced 3.0 Tesla breast MRI. Initially, MRI scans were read by radiologists in a routine clinical setting. All histologically confirmed index lesions were re-evaluated by two dedicated breast radiologists. Sensitivity and specificity for the three MRI readings were determined, and the diagnostic value of breast MRI in addition to conventional imaging was assessed. Interobserver reliability between the three readings was evaluated.

**Results:**

MRI examinations of 207 patients were analyzed. Seventy-eight of 207 (37.7%) patients had a malignant lesion, of which 33 (42.3%) patients had pure DCIS and 45 (57.7%) invasive breast cancer. Sensitivity of breast MRI was 66.7% during routine, and 89.3% and 94.7% during expert reading. Specificity was 77.5% in the routine setting, and 61.0% and 33.3% during expert reading. In the routine setting, MRI provided additional diagnostic information over clinical information and conventional imaging, as the Area Under the ROC Curve increased from 0.76 to 0.81. Expert MRI reading was associated with a stronger improvement of the AUC to 0.87. Interobserver reliability between the three MRI readings was fair and moderate.

**Conclusions:**

3.0 T breast MRI of nonpalpable breast lesions is of added diagnostic value for the diagnosis of *in situ* and invasive breast cancer.

## Introduction

Dynamic contrast-enhanced magnetic resonance imaging (MRI) of the breast has become mainstream for the detection and characterization of breast lesions in clinical practice [1]–[3]. Current indications for breast MRI include screening of high-risk populations, monitoring of the treatment response in patients receiving neoadjuvant chemotherapy, tumor detection in patients with metastases of an unknown primary, and evaluation of silicone breast prostheses [4], [5]. Two randomised controlled trials have studied the impact of preoperative breast MRI on the reoperation rate after breast-conserving surgical treatment [6], [7]. In these studies, the addition of breast MRI to conventional imaging did not reduce the number of additional surgical interventions, e.g. repeat lumpectomy or mastectomy. Preoperative breast MRI is therefore currently only indicated in a selection of patients, e.g. in patients with invasive lobular carcinoma, patients with a discrepancy in lesion size of more than 1 cm between mammography and ultrasound, and patients eligible for partial breast irradiation, in whom the clinical benefit of preoperative breast MRI is more clear [8].

Several studies assessed the overall diagnostic accuracy of breast MRI in patients with suspicious breast lesions, reporting a high sensitivity of around 90%, and a considerably lower specificity of 70–75% [2], [9], [10]. However, few articles addressed the diagnostic value of MRI in addition to conventional imaging (i.e. mammography and ultrasound) [11], [12]. Furthermore, most breast MRI studies were performed using a 1.5 Tesla (T) MRI system [10]. Imaging at higher field strength may be beneficial because of the higher signal-to-noise ratio (SNR). Only one relatively small study reported a higher diagnostic accuracy for breast MRI at 3.0 T compared to 1.5 T [13]. To our knowledge, large studies assessing the overall diagnostic performance of breast MRI at 3.0 T are lacking.

The aim of this study was to assess the added diagnostic value of 3.0 Tesla breast MRI in patients with nonpalpable breast disease who were referred for histological biopsy. In addition, we evaluated interobserver variability between routine and expert reading for the evaluation of 3.0 T breast MRI.

## Materials and Methods

### Patients

This study was conducted using data from the MONET study (**M**R mammography **O**f **N**onpalpable Br**E**ast **T**umors), a multicenter randomized controlled trial (NCT00302120) designed to assess the impact of dynamic contrast-enhanced breast MRI on the re-excision rate of women with nonpalpable breast lesions. The study protocol was approved by the institutional review boards of the University Medical Center Utrecht, Diakonessen Hospital Utrecht, and the Albert Schweitzer Hospital, and written informed consent was obtained from all patients. Detailed methods were described elsewhere [6]. Briefly, between January 2006 and May 2009, 463 patients with nonpalpable breast lesions classified as BI-RADS 3-5 on mammography or ultrasound with an indication for histological biopsy, were randomly allocated to routine clinical care or to routine clinical care with an additional 3.0 T breast MRI. Mammography and breast ultrasound were read in a routine clinical setting by several radiologists at the center of patient inclusion. In the present study, we included only patients who were randomized to undergo additional breast MRI. Histological analysis was performed and lesions were classified as benign or malignant (i.e. ductal carcinoma in situ or invasive carcinoma) based on biopsy (in case of a benign lesion) or the surgical specimen. Only the index lesions (i.e. the nonpalpable BI-RADS 3–5 lesion for which patients were included) were included in the analyses.

### MR imaging

MR imaging was performed prior to large-core needle biopsy (LCNB). All breast MRI scans were performed on a 3.0 T clinical MR system (Achieva, Philips Healthcare, Best, the Netherlands) at the University Medical Centre Utrecht. The system employs gradient amplitudes up to 80 mT/m and slew rates up to 200 mT/m/ms. Patients were placed in prone position on a dedicated, four-channel phased-array bilateral breast coil (MRI devices, Würzburg, Germany). All series were acquired using SENSE parallel imaging techniques. The scan protocol included a transverse, dynamic contrast-enhanced fat-suppressed T1-weighted gradient echo series (TE/TR 1.3/3.4 ms; flip angle 10°; FOV 320×320 mm^2^, acquired voxel size 0.91×0.91×2.00 mm^3^, reconstructed voxel size 0.83×0.83×1.00 mm^3^; dynamic scan duration 60 sec). For the contrast-enhanced series, fat suppression was employed using SPAIR fat suppression. One scan was acquired before, and five scans were acquired immediately after administration of 0.1 mmol/kg Gadolinium-DTPA (Magnevist, Schering, Germany). Also, a transverse high-resolution fat-suppressed T1-weighted fast gradient echo series (TE/TR 1.7/4.5 ms; inversion delay SPAIR 130 ms; flip angle 10°; FOV 340×340 mm^2^, acquired voxel size 0.66×0.66×1.6 mm^3^, reconstructed voxel size 0.66×0.66×0.80 mm^3^) and a fat-suppressed T2-weighted spin echo series (TE/TR 120/9022 ms; inversion delay SPAIR 125 ms; flip angle 90°; FOV 340×340 mm^2^, acquired voxel size 1.01×1.31×2.0 mm^3^, reconstructed voxel size 0.66×0.66×2.00 mm^3^) were acquired.

### Image analysis

During the MONET study, MRI examinations were initially read by four breast radiologists in a routine clinical setting according to the BI-RADS MRI lexicon as proposed by the American College of Radiology [14]. For this present study, all index lesions were re-evaluated by two trained and dedicated breast radiologists in a review setting. They both had about seven years experience in reading breast MRI and were not involved in routine reading. According to the BI-RADS MRI lexicon, lesions were classified as focus, mass, or non-mass like enhancement. Subsequently, the corresponding features (i.e. shape, margin and mass enhancement for mass lesions, distribution modifiers and internal enhancement for non-mass lesions) were assessed. During expert MRI reading, information on the mammography images was provided. Expert readers were blinded for histological results and a software tool (CADstream, Confirma, Chicago, Illinois) for image post-processing was used for a standardized analysis and interpretation of the dynamic contrast-enhanced images. CADstream is a commercially available computer-aided detection system. Color overlays on the dynamic MR images were used to indicate the threshold of initial enhancement. Furthermore, the color overlay allows differentiation between the three types of enhancement (persistent, plateau, and washout) in the late phase after contrast injection. Expert readers were allowed to exclude MRI examinations if, to their opinion, the image quality was insufficient for analysis.

### Statistics

Univariate analysis was performed to assess differences between patients with benign and malignant lesions. Continuous variables were analysed using the independent sample T-test. For categorical variables, differences in proportions were tested using the Pearson's chi-squared test or the Fisher's exact test. Clinical, mammographic and ultrasound features that were most significantly associated with malignancy were introduced into a first model. Three other models were constructed after the addition of the three MRI readings. For every ten malignancies, one determinant was allowed to be included in the logistic regression model. Discrimination between benign and malignant lesions was estimated by the Area Under the Receiver Operating Characteristic (ROC) Curve (AUC). Differences between AUCs were tested according to Hanley and McNeil [15]. Calibration was measured using the Hosmer-Lemeshow goodness-of-fit test. To calculate sensitivity and specificity of the three MRI readings, BI-RADS classifications of 1 and 2 were considered as negative, and BI-RADS 3–5 as positive test results. The same cut-off value (i.e., BI-RADS 1 and 2 for benign lesions and BI-RADS 3–5 for malignant lesions) was used to calculate the reliability using κ statistics. In addition, interobserver agreement, defined as the degree to which ratings are identical (the measurement error) was calculated using the proportion of agreement [16]. Differences were tested between all three MR readings (routine reading, expert reader 1 and 2). A κ value below 0.20 indicated poor agreement; 0.21–0.40 fair agreement; 0.41–0.60 moderate agreement; 0.61–0.80 good agreement; and a κ value of 0.81–1.00 indicated very good agreement [17]. Statistical analyses were performed using the software packages SPSS (version 20.0, Chicago, Illinois).

## Results

### Patients

MRI examinations of 207 patients were analyzed. The mean age of patients was 55.1 years and 60.2% of patients were referred by the national breast cancer screening program (Table 1). Mammography showed microcalcifications only in 121/207 (58.5%) patients. LCNB showed a malignant lesion in 78/207 (37.7%) patients. Thirty-three of 78 (42.3%) patients were diagnosed with pure ductal carcinoma in situ and 45/78 (57.7%) with invasive breast cancer, with or without an in situ component.

**Table 1:** Baseline table presenting clinical patient characteristics and features on mammography and ultrasound for the 207 index lesions.

	Benign (%)	Malignant (%)	p-value	Total (%)
**Clinical characteristics**				
Number of patients	129 (62.3)	78 (37.7)		207 (100)
Age in years, mean ± SD (n = 207)	52.9±9.8	58.7±7.6	0.00*	55.1±9.5
BMI in kg/m^2^, mean ± SD (n = 201)	25.0±3.8	26.2±3.8	0.026*	25.4±3.8
Breast cancer in first degree relative (n = 204)	19 (15.1)	24 (30.8)	0.008∧	43 (21.1)
Presence of clinical symptoms (n = 204)	42 (33.3)	16 (20.5)	0.049∧	58 (28.4)
Detected in screening program (n = 201)	65 (51.6)	56 (74.4)	0.001∧	121 (60.2)
**Mammography**				
**Type of finding (n = 207)**				
Microcalcifications only	80 (62.0)	41 (52.6)	0.37∧	121 (58.5)
Mass lesion (with/without microcalcifications)	39 (30.2)	28 (35.9)		67 (32.4)
Other	10 (7.8)	9 (11.5)		19 (9.2)
**BI-RADS classification (n = 207)**				
BI-RADS 1 or 2 a	8 (6.2)	0 (0.0)	0.000^&^	8 (3.9)
BI-RADS 3	56 (43.4)	23 (29.5)		79 (38.2)
BI-RADS 4	64 (49.6)	45 (57.7)		109 (52.7)
BI-RADS 5	1 (0.8)	10 (12.8)		11 (5.3)
**Ultrasound**				
Performed (n = 207)	94 (72.9)	67 (85.9)	0.029∧	161 (77.8)
**Type of finding (n = 161)**				
No lesion	57 (60.6)	31 (46.3)	0.19∧	88 (54.7)
Solid lesion	28 (29.8)	28 (41.8)		56 (34.8)
Other b	9 (9.6)	8 (11.9)		17 (10.6)
**BI-RADS classification (n = 160)**				
BI-RADS 1 or 2	63 (67.7)	34 (50.7)	0.000∧	97 (60.6)
BI-RADS 3	19 (20.4)	6 (9.0)		25 (15.6)
BI-RADS 4	10 (10.8)	16 (23.9)		26 (16.3)
BI-RADS 5	1 (1.1)	11 (16.4)		12 (7.5)

*independent sample T-test, ∧chi-square test, ^&^Fisher's exact test

a occult or benign lesion on mammography (BI-RADS 1 or 2), classified as BI-RADS 3, 4 or 5 on ultrasound.

b cystic lesions, hypoechoic areas not otherwise specified, and areas of architectural distortion.

### Diagnostic performance of MRI

With routine MRI reading, 15/31 (48.4%, 95% CI 32.0–65.2) of BI-RADS 3 lesions, 19/31 (61.3%, 95% CI 43.8–76.3) of BI-RADS 4 lesions, and 18/19 (94.7%, 95% CI 75.4–99.1) of BI-RADS 5 lesions were proven malignant by histology. Table 2 shows the different BI-RADS classifications of routine and expert readers versus the histological outcome. For routine reading, the sensitivity of MRI for the detection of malignancy was 66.7% and specificity was 77.5% (Table 3). Thus, in 26/78 (33.3%) patients, the malignant lesion was not detected on MRI; in 21/26 (80.8%) patients this concerned pure DCIS without an invasive component.

**Table 2:** BI-RADS MRI classifications for routine and expert MRI reading.

	Benign (%)	In situ (%)	Invasive (%)	Total (%)
Routine reading				
**BI-RADS classification (207)**				
BI-RADS 1/2	100 (77.5)	21 (63.6)	5 (11.1)	126 (60.9)
BI-RADS 3	16 (12.4)	6 (18.2)	9 (20.0)	31 (15.0)
BI-RADS 4	12 (9.3)	6 (18.2)	13 (28.9)	31 (15.0)
BI-RADS 5	1 (0.8)	0 (0.0)	18 (40.0)	19 (9.2)
**Expert reader 1**				
**BI-RADS classification (198)**				
BI-RADS 1/2	75 (61.0)	8 (26.7)	0 (0.0)	83 (41.9)
BI-RADS 3	24 (19.5)	7 (23.3)	5 (11.1)	36 (18.2)
BI-RADS 4	24 (19.5)	15 (50.0)	21 (46.7)	60 (30.3)
BI-RADS 5	0 (0.0)	0 (0.0)	19 (42.2)	19 (9.6)
**Expert reader 2**				
**BI-RADS classification (192)**				
BI-RADS 1/2	39 (33.3)	4 (12.9)	0 (0.0)	43 (22.4)
BI-RADS 3	34 (29.1)	4 (12.9)	3 (6.8)	41 (21.4)
BI-RADS 4	44 (37.6)	21 (67.7)	34 (77.3)	99 (51.6)
BI-RADS 5	0 (0.0)	2 (6.5)	7 (15.9)	9 (4.7)

**Table 3:** Sensitivity and specificity for routine and expert reading of 3.0 T breast MRI.

	Sensitivity (95% CI)	Specificity (95% CI)	AUC (95% CI)
**Routine reading**	66.7 (55.6–76.1)	77.5 (69.6–83.9)	0.81 (0.75–0.88)
**Expert reader 1**	89.3 (80.3–94.5)	61.0 (52.2–69.1)	0.87 (0.82–0.92)
**Expert reader 2**	94.7 (87.0–97.9)	33.3 (25.4–42.3)	0.87 (0.81–0.92)

Expert reader 1 judged nine MRI examinations to be of insufficient image quality for assessment. These included three pure DCIS and six benign lesions. Positive predictive values were 33.3% (95% CI 20.2–49.7), 60.0% (95% CI 47.4–71.4), and 100% (95% CI 83.2–100.0) for BI-RADS 3, 4, and 5 lesions, respectively. Sensitivity was 89.3% and specificity was 61.0%. In 8/75 (10.7%) of patients, the malignant lesion (pure DCIS in all cases) was not seen on MRI.

**Figure 1:**
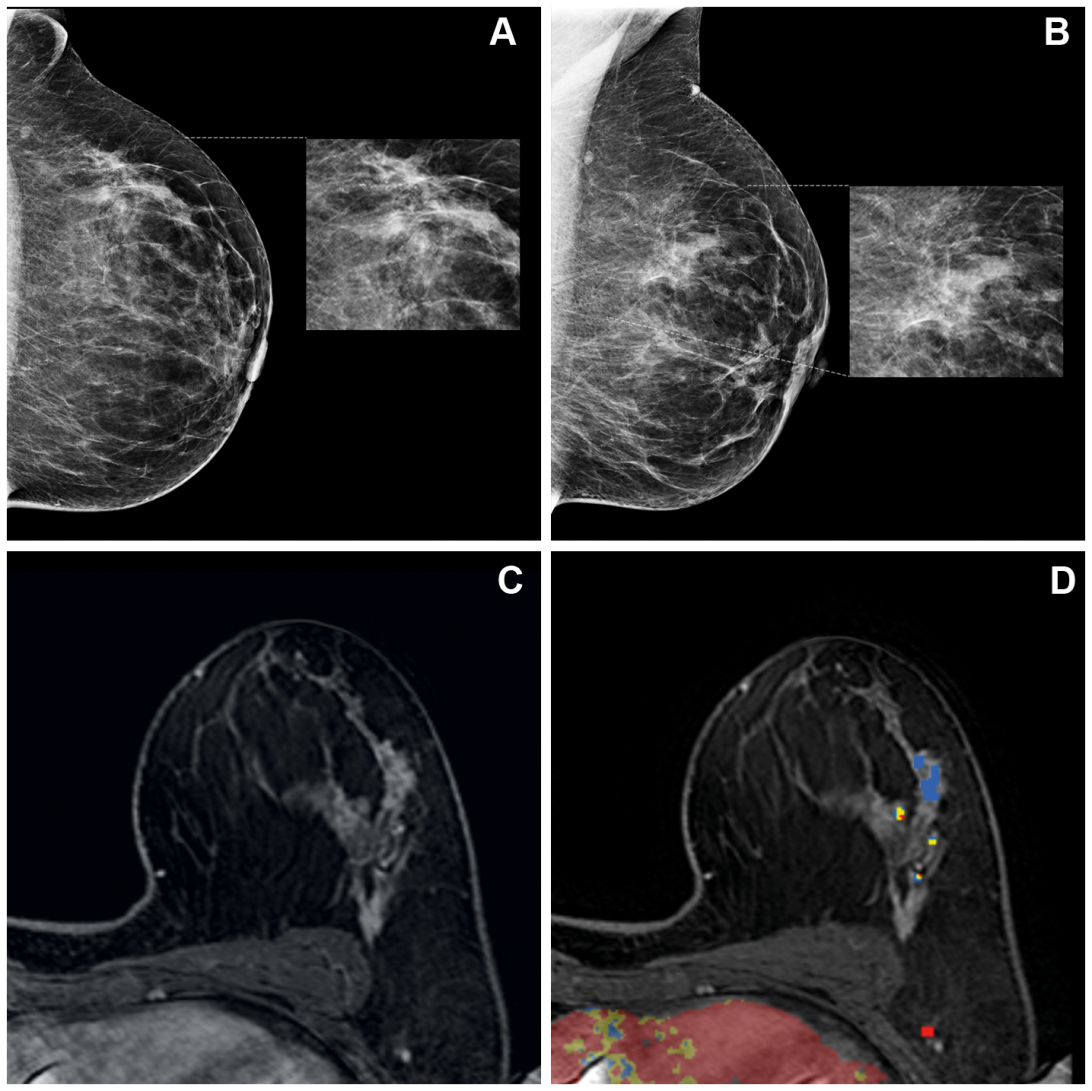
Craniocaudal (a) and mediolateral oblique (b) mammogram of a 52 year old patient with BI-RADS 5 microcalcifications in the left lateral upper quadrant. Ultrasound imaging was unremarkable (BI-RADS 1). During routine reading, the MRI examination was classified as BI-RADS 4. Both expert reader 1 and 2 classified the lesion as an area of non-mass like enhancement with clumped internal enhancement and a segmental distribution. Kinetics showed a rapid initial rise and a plateau stage during the delayed phase. A BI-RADS 4 and 5 classification was given by expert reader 1 and 2, respectively. Figure 1 shows the dynamic contrast-enhanced MRI (c) and the MR image imported in the CAD software (d). The color-coded overlay indicates the type of enhancement after contrast injection in the late phase. Red, yellow and blue illustrate a washout-, plateau- and persistent- enhancement curve, respectively. Stereotactic biopsy and surgery both showed DCIS without an invasive component.

**Figure 2:**
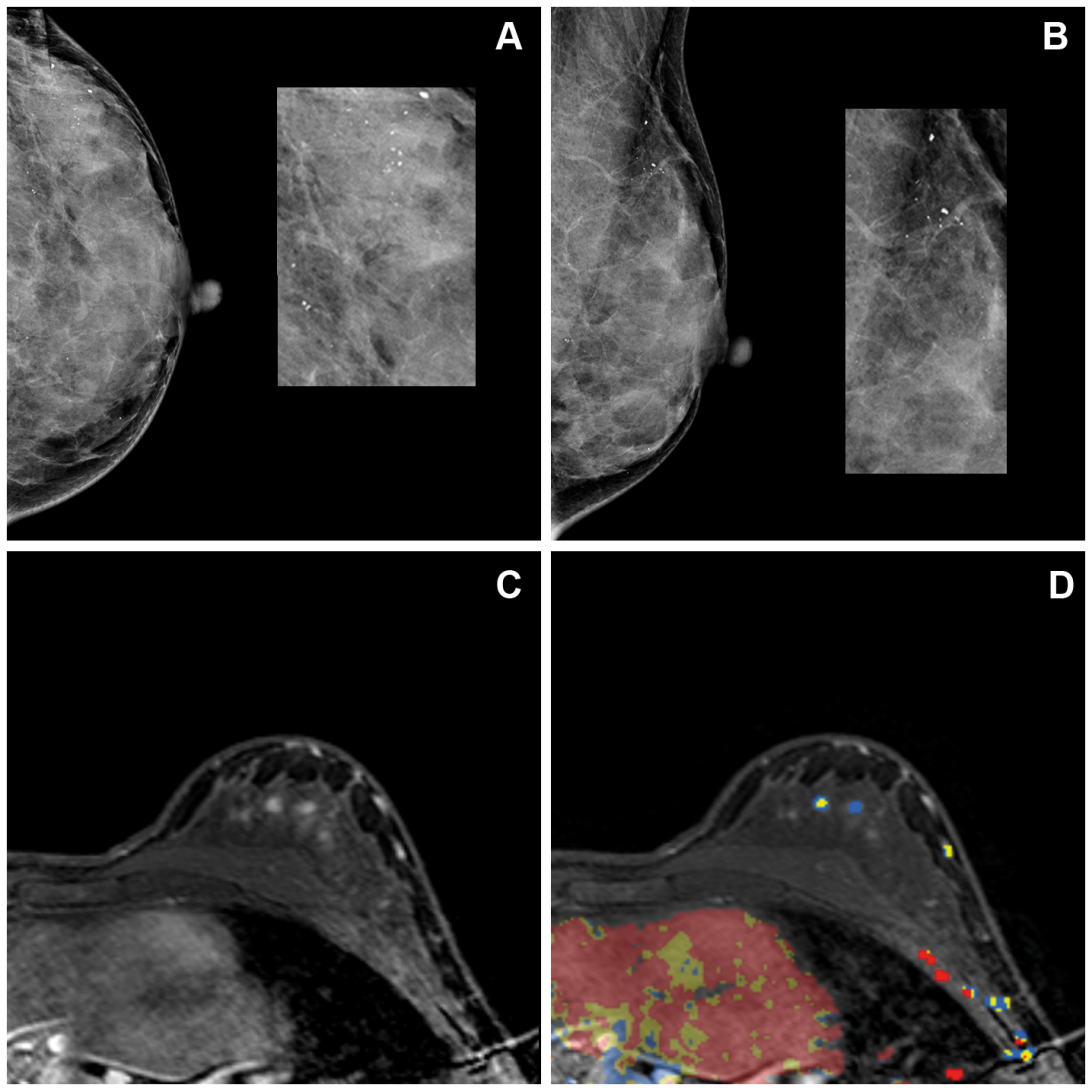
Craniocaudal (a) and mediolateral oblique (b) mammogram of a 40 year old, asymptomatic woman underwent mammography during follow-up after right-sided breast cancer, for which she underwent mastectomy. Mammography showed BI-RADS 4 microcalcifications in the lateral upper quadrant of the left breast. Ultrasound imaging was unremarkable (BI-RADS 1). During routine MRI reading, a BI-RADS 1 classification was assigned. Expert reader 1 reported an area of non-mass-like enhancement with a diffuse distribution, heterogeneous internal enhancement and classified MR imaging as BI-RADS 4. In addition, expert reader 2 described an area of non-mass-like enhancement with a segmental distribution and clumped internal enhancement, and reported a BI-RADS 4. Kinetics showed a rapid initial rise and a plateau stage during the delayed phase. Figure 2 shows the dynamic contrast-enhanced MRI (c) and the MR image imported in the CAD software (d). The color-coded overlay indicates the type of enhancement after contrast injection in the late phase. Yellow and blue illustrate a plateau- and persistent- enhancement curve, respectively. Stereotactic biopsy showed normal breast tissue with minor fibrocystic changes and the extensive presence of microcalcifications.

Expert reader 2 rated fourteen MRI examinations of insufficient image quality, and one MRI examination was not analyzed unintentionally. These were three malignant and twelve benign lesions. Positive predictive values were 17.1% (95% CI 8.5–31.3), 55.6% (95% CI 45.8–65.0) and 100% (95% CI 70.1–100.0) for lesions classified as BI-RADS 3, 4 and 5, respectively. Sensitivity was 94.7% and specificity was 33.3%. In 4/75 (5.3%) of patients, the malignant lesion (pure DCIS in all cases) was not seen on MRI. Figure 1 and 2 show some typical examples of mammography and MR imaging in two patients.

### Added diagnostic value of 3.0 T MR imaging

**Figure 3:**
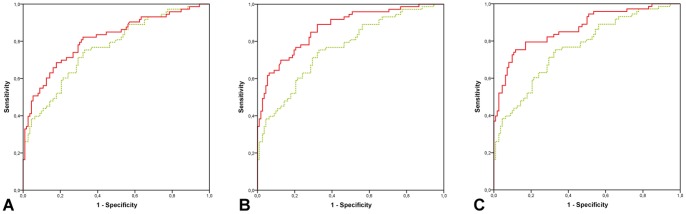
ROC analysis shows an AUC of 0.76 for the model with clinical characteristics and conventional imaging, which is displayed as the green, dashed line in the three graphs (a-c). The red, solid lines show the AUC's for the models after addition of MRI reading. The model for routine reading has an AUC of 0.81 (a), both models with expert MRI reading have an AUC of 0.87 (b-c).

The logistic regression model with clinical characteristics (i.e., age and breast cancer in first degree relatives) and conventional imaging (i.e., mammography and ultrasound) had an AUC of 0.76 (95% CI 0.69–0.83). In the routine setting, MRI was of added diagnostic value over clinical characteristics and conventional imaging: after the addition of routine MRI reading to the regression model, the AUC increased to 0.81 (95% CI 0.75–0.88) with a p-value <0.05. Expert MRI reading was associated with an even stronger improvement in AUC, from 0.76 (95% CI 0.69–0.83) to 0.87 (95% CI 0.82–0.92) for reader 1 and 0.87 (95% CI 0.81–0.92) for reader 2. The differences between the AUC's with and without the addition of MRI were statistically significant for both expert readers (p-value <0.001). All models showed good calibration (p>0.1) (Figure 3).

### Interobserver reliability and agreement

The proportion of agreement between the three observers varied between 56.8 and 78.8% for the three MRI readings (Table 4). Reliability between routine reading and expert reader 1 (kappa 0.59) and between expert reader 1 and expert reader 2 (kappa 0.48) was moderate. The reliability between routine reading and expert reader 2 was fair (kappa of 0.22).

**Table 4:** The interobserver reliability (κ statistics) and agreement (proportion of agreement) for BI-RADS MRI classifications.

	κ statistics (95% CI)	Agreement (95% CI)
**Routine reading vs. expert reader 1**	0.59 (0.48–0.69)	78.8 (72.6–83.9)
**Routine reading vs. expert reader 2**	0.22 (0.12–0.31)	56.8 (49.7–63.6)
**Expert reader 1 vs. expert reader 2**	0.48 (0.36–0.59)	76.1 (69.5–81.6)

## Discussion

Our results show that 3.0 Tesla breast MRI of nonpalpable lesions is of added diagnostic value in the diagnosis of in situ and invasive breast cancer. Both during routine and expert reading, addition of breast MRI allowed better discrimination between benign and malignant disease, with AUC's increasing from 0.76 to 0.81 with routine MRI reading and from 0.76 to 0.87 for expert reading. Although many studies assessed the sensitivity and specificity of breast MRI in itself, only a few studies have looked at the added value of breast MRI when combined with mammography and ultrasound. Berg et al. described that the combination of mammography, clinical examination and MR imaging was more sensitive (99.4%) for invasive cancer than any other test or combination of different tests [11]. Malur et al. reported a sensitivity of 99.4% when combining mammography, ultrasound and MR imaging [12]. No studies have assessed the added value of breast MRI over conventional imaging in patients with nonpalpable breast lesions. Due to the introduction of breast cancer screening programs, the detection of clinically occult, often nonpalpable, suspicious lesions on mammography has increased over the years [18]. A large percentage of these patients present with microcalcifications on mammography. Ruling out DCIS in these patients can be a diagnostic challenge: mammography has a high sensitivity for the detection of microcalcifications, but also leads to many biopsies in benign cases [19], [20].

In this study, sensitivity was 66.7% and specificity was 77.5% for routine reading of breast MRI. For the two expert readers, these sensitivities increased to 89.3% and 94.7%. This increase in sensitivity was accompanied with a decrease in specificity to 61.0% and 33.3%. The low specificity of 33.3% of expert reader 2 was caused by the high percentage of benign lesions, which were classified as BI-RADS 3 (29.1%) and BI-RADS 4 (37.6%). This reader had, however, the highest sensitivity among all readings. Next to the assessment of sensitivities and specificities, we specifically investigated the diagnostic performance of the different imaging methods using ROC analyses. In clinical practice, the BI-RADS lexicon is used to classify lesions in categories with different risks on malignancy. For calculating measures of diagnostic performance (i.e. sensitivity and specificity), a certain cut-off point must be chosen. The area under the ROC curve however is able to show the diagnostic performance irrespective of a cut-off point, and therefore reflects the overall discriminative value of various tests [21].

In this study, MR images were read following the BI-RADS-MRI lexicon, using T1 and T2 weighted series for morphological analysis of the lesion and dynamic contrast-enhanced series for analysis of the enhancement pattern of the lesions over time. This yields a proven high sensitivity for the characterization of benign versus malignant breast lesions [5]. Currently, other promising MR techniques such as diffusion weighted imaging and MR spectroscopy are becoming more widely available, which may further improve the diagnostic accuracy of breast MRI [22]. Furthermore, the results of this study can probably be extrapolated to studies performed at 1.5 T imaging, because of the comparable results of breast MRI at 3.0 T and 1.5 T MRI [13]. Our study, however, is one of the few studies which has evaluated a relatively large patient population with small breast lesions on a 3.0 T MRI system.

Both the COMICE and the MONET trial did not show an added value of breast MRI for the surgical outcome of breast cancer patients [6], [7]. Turnbull et al. reported no reduction in the reoperation rate after the addition of breast MRI. Both in the MRI group and in the control group, the percentage of patients requiring reoperation was 19% [7]. The MONET study showed even a higher percentage of re-excisions in the MRI group (34%) versus the control group (12%) [6]. In this study, we only report on the diagnostic performance of breast MRI. Unfortunately, it is not feasible to repeat the analyses for the therapeutic outcome of this trial for the two expert readers. However, while preoperative MRI nowadays is only used in a selection of patients, our results show that MRI does have additional value over baseline characteristics and conventional imaging in the evaluation of index lesions in the breast. This effect might even be underestimated, because in the current study, only clinically occult (nonpalpable) lesions were included.

We observed a difference in the diagnostic performance between routine reading during the initial study and expert reading afterwards. This difference can be explained by several reasons. First, the MONET study was performed shortly after the introduction of breast MRI in clinical practice. Through the years, more knowledge about MRI reading was acquired, especially in the assessment and evaluation of lesions showing non-mass-like-enhancement. A large percentage of malignancies in this study consisted of pure DCIS (42.3%). Rosen et al. report that pure DCIS in 59.4% of the cases is visualized as non-mass-like-enhancement on breast MRI [23]. Differentiation between benign and malignant is challenging in these lesions, because of the absence of the typical malignant wash-out pattern and poorly defined boundaries in non-mass-like lesions [24]–[26]. Second, during expert reading, all breast MRI examinations were analyzed using computer aided diagnosis (CAD) software (CADstream, Confirma, Chicago, Illinois), a dedicated software tool for the automatic analysis of breast lesions. The diagnostic performance has been shown to improve when readers use a dedicated CAD system for dynamic breast MRI analysis [27], [28]. Third, the expert readers were allowed to exclude MRI examinations if, to their opinion, the quality of the exam was not sufficient to read. Also this can partly explain the difference between routine and expert reading. We observed fair to moderate interobserver agreement between the three MRI readings. Previous studies have shown the presence of significant interobserver variation and a considerable learning curve in the interpretation of breast MRI [29].

This study has a some limitations. Sensitivity and specificity for mammography and ultrasound were not described, because the study population consisted of patients with BI-RADS 3-5 lesions on mammography or ultrasound only. To assure that histopathological confirmation was available for all lesions, we only assessed the index lesions of which LCNB was performed. Any potential multifocal, multicentric or contralateral lesions were not included in the analyses, while breast MRI is currently often used to detect multifocal or multicentric disease [5]. In this study, the BI-RADS MRI cut-off value for malignancy was set at BI-RADS 3, because in our hospital, many BI-RADS 3 patients are referred for biopsy. This, however, resulted in a lower specificity. Another shortcoming of our study was the fact that MR readings (both routine and expert reading) were performed by different radiologists than reading of conventional imaging. Furthermore, MRI reading in a review setting can induce bias by over reading, which may have resulted in the lower specificity we found during expert reading. Finally, we cannot draw any conclusions from a therapeutic perspective. This study exclusively reports on the diagnostic performance of 3.0 Tesla breast MRI. However, our results indicate that the evaluation and handling of information on breast MRI in clinical practice may not be optimal.

In conclusion, this study indicates that 3.0 T breast MRI of nonpalpable breast lesions is of added diagnostic value in the diagnosis of in situ and invasive breast cancer. More evidence, however, is needed to support this conclusion.
